# Joint hypermobility in girls with idiopathic scoliosis: relation with age, curve pattern and curve size

**DOI:** 10.1186/1748-7161-8-S2-P2

**Published:** 2013-09-18

**Authors:** Dariusz Czaprowski, Tomasz Kotwicki, Paulina Pawłowska, Łukasz Stoliński, Mateusz Kozinoga, Piotr Janusz

**Affiliations:** 1 Department of Physiotherapy, Józef Rusiecki University College, Olsztyn, Poland; Rehasport Clinic, Poznań, Poland

## Background

A complete musculoskeletal examination should include the specific tests to detect hypermobile individuals, especially when physiotherapy affecting joint mobility is planned. Joint hypermobility (JH) is defined as an excessive range of motion of joints, taking into consideration the subject’s gender, age, and ethnic background[1].

## Purpose

The goal of this study was to assess the prevalence of JH in girls with idiopathic scoliosis (IS), taking into account the age, curve pattern and curve size. 

## Methods

The study group included 147 Caucasian girls with IS, aged 9-18 years (mean 13.6 ±2.2), Cobb angle range 11°-65°, mean 27.9 ±11.9, comprising 42 single thoracic, 31 single lumbar and 74 double curve scoliosis. According to the Cobb angle, there were 70 mild (10-24º), 54 moderate (25-40º) and 23 severe (> 45º) curves. The control group included 147 girls aged 9-18 years (mean 13.1 ±.3), selected at random from the group of 300 girls free of IS (angle of trunk rotation <5°). The presence of JH was assessed with the 9-point Beighton scale [1], using the cut-off ≥5 points.

## Results

JH was diagnosed in 24.5% of IS girls, whilst in the control group, it was diagnosed in 15.1% (p=0.04). The prevalence of JH was significantly (p=0.03) lower in IS girls aged 16-18 years in comparison to younger individuals (9-15). There was no difference regarding JH occurrence among girls with mild, moderate and severe scoliosis (p=0.8). No significant differences in JH prevalence was observed among girls with single thoracic, single lumbar and double curve scoliosis (p=0.68). The number of vertebrae within curvature did not influence the prevalence of JH (p=0.13). 

## Conclusions and discussion

JH appeared more often in IS girls than in healthy controls. Its prevalence decreased with age. No relation between JH prevalence and curve pattern, curve size or number of vertebrae within curvature was found.
